# Evaluation of *Bacillus aryabhattai* B8W22 peroxidase for phenol removal in waste water effluents

**DOI:** 10.1186/s12866-023-02850-9

**Published:** 2023-04-29

**Authors:** Alaa Elmetwalli, Nanis G. Allam, Mervat G. Hassan, Aisha Nawaf Albalawi, Azza Shalaby, Karim Samy El-Said, Afrah Fatthi Salama

**Affiliations:** 1 Department of Clinical Trial Research Unit and Drug Discovery, Egyptian Liver Research Institute and Hospital (ELRIAH), Mansoura, Egypt; 2 Microbiology Division, Botany Department, Faculty of Science, Tanta University, Tanta, 31527, Egypt; 3 Department of Botany and Microbiology, Faculty of Science, Benha University, Benha, 33516 Egypt; 4 Department of Biology , University of Haql College, University of Tabuk, Tabuk, 71491, Saudi Arabia; 5 Biochemistry Division, Chemistry Department, Faculty of Science, Tanta University, Tanta, 31527, Egypt

**Keywords:** *Bacillus aryabhattai* B8W22, Peroxidase Enzyme activity, Phenol degradation, Wastewater

## Abstract

Environmental contamination by phenol has been reported in both aquatic and atmospheric environments. This study aimed to separate and purify the peroxidase enzyme from bacteria that degrade phenol from wastewater sources. An enrichment culture of MSM was used to screen 25 bacterial isolates from different water samples for peroxidase production, six of the isolates exhibited high levels of peroxidase enzyme activity. Qualitative analysis of peroxidase revealed that isolate No. 4 had the highest halo zones (Poly-R478: 14.79 ± 0.78 mm, Azure B: 8.81 ± 0.61 mm). The promising isolate was identified as *Bacillus aryabhattai* B8W22 by 16S rRNA gene sequencing with accession number OP458197. As carbon and nitrogen sources, mannitol and sodium nitrate were utilized to achieve maximum peroxidase production. A 30-h incubation period was used with pH 6.0, 30 °C, mannitol, and sodium nitrate, respectively, for maximal production of peroxidase. Purified peroxidase enzyme showed 0.012 U/mg specific activity, and SDS-PAGE analysis indicated a molecular weight of 66 kDa. The purified enzyme exhibits maximum activity and thermal stability at pH values of 4.0 and 8.0, respectively, with maximum activity at 30 °C and complete thermal stability at 40 °C. In the purified enzyme, the *Km* value was 6.942 mg/ml and the *Vmax* value was 4.132 mol/ml/hr, respectively. The results demonstrated that *Bacillus aryabhattai* B8W22 has promising potential for degrading phenols from various phenol-polluted wastewater sources.

**Supplementary Information:**

The online version contains supplementary material available at 10.1186/s12866-023-02850-9.

## Introduction

Water supplies are essential for maintaining a healthy environment and a sufficient food supply for all living creatures. As human populations and economies have increased, the demand for freshwater globally has been rising quickly [1]. Phenolic compounds are among the most persistent harmful organic pollutants found in wastewater effluents and discharges from chemical process industries such as pulp and paper, pharmaceuticals, agrochemicals, petrochemicals, and pesticide manufacturing which are resistant to environmental degradation through chemical, biological, and photolytic processes [2]. The US Environmental Protection Agency has classified phenol as one of 126 priority pollutants also phenol has been related to environmental contamination in both the aquatic and atmospheric realms. Furthermore, phenol spills were the primary source of aquatic contamination [3]. Because of their toxicity, ability to alter the endocrine system, and carcinogenic properties, the accumulation of these pollutants can be harmful to human health [4]. As a result, poor handling and disposal of these carcinogenic hazardous chemicals constitute a serious threat to the environment and ecology [5].

Biodegradation, which is both environmentally friendly and cost-effective, is a viable approach for removing phenol [6]. The peroxidases are enzymes capable of catalyzing the oxidative coupling reactions of various phenolic compounds in the presence of H_2_O_2_ [7]. The oxidative dehalogenation of pentachlorophenol to totetrachloro-1,4-benzoquinone by various peroxidases from *Phanerochaete chrysosporium*, myeloperoxidases, lactoperoxidases, and chloroperoxidases from *Caldariomyces fumago* could be achieved by peroxidases [8]. In addition, extracellular manganese peroxidase was detected in *P. chrysosporium*, *P. sordida*, and *C. subvermispora*. By oxidizing Mn^2+^ into Mn^3+^, that extracellular peroxidase undergoes two-electron oxidation by H_2_O_2_, which in turn oxidizes phenolic compounds [9].

As yet, numerous bacteria that degrade phenol allied with *Pseudomonas*, *Acinetobacter*, *Halomonas*, and *Bacillus* sp*.*, etc., have been sequestered from non-extreme settings, for instance, neutral pH conditions [10]. The *Bacillus* sp., in particular, has been recovered from a variety of habitats and is capable of degrading phenol via ortho and meta catechol routes at a variety of temperatures, pH, and concentrations [11]. Peroxidase synthesis has been reported by numerous bacteria, mostly, *actinobacteria* [12] and *Bacillus* sp. [13]. Furthermore, bacteria seem to have more hope for increasing peroxidase production. This may be due to their gene flexibility comparing to fungi, while fungi's genes are not.

*Bacillus* sp. is described as an "important workhorse industrial microbes" with "enhanced enzyme production capacity." They grow quickly and can make a lot of proteins outside of their cells. Additionally, several *Bacillus* sp. had been utilized to produce pectinolytic and cellulolytic enzymes [14]. Peroxidase production in plants and fungi has been extensively researched [15].

Peroxidases are oxidoreductases of great interest due to their high redox potentials and ability to oxidize compounds that are known to be resistant to degradation. The peroxidases convert phenol into phenoxy radicals, which produce polymeric precipitates after hydrogen peroxide activates them [16, 17]. Illustratively, peroxidases work by oxidizing enzymes with hydrogen peroxide, which initiates the oxidation of two molecules of phenol [18]. In this case, assuming that the formed dimer is insoluble, the stoichiometric ratio between the peroxide consumed and the precipitated phenolic compound is equal to 1:2. As a result of a sequence of reactions between soluble dimers and the enzyme, larger polymers were produced, resulting in the theoretical stoichiometry being different from the measured value [19]. Water-insoluble coupling products can be utilized as phenolic substrates and further transformed into trimmers, tetramers, or larger polymers. Accordingly, as polymer sizes increase during the polymerization reaction, the [H_2_O_2_]/[phenol] ratios tend towards unity [19]. Collectively, there are four steps in the catalytic polymerization cycle of peroxidases:

1 $$\mathrm{Peroxidase }+ {\mathrm{H}}_{2}{\mathrm{O}}_{2} \to \mathrm{ C}1 + {\mathrm{H}}_{2}\mathrm{O}$$

2 $$\mathrm{Ci}+\mathrm{ Peroxidase\ substrate }\to \mathrm{ Cii }+\mathrm{ free\ radical\ product}$$

3 $$\mathrm{Cii }+\mathrm{ Peroxidase\ substrate }\to \mathrm{ Peroxidase }+\mathrm{ free\ radical\ product }+ {\mathrm{H}}_{2}{\mathrm{O}}_{2}$$

4 $$\mathrm{Free\ radical\ product }+\mathrm{ free\ radical\ product }+ {\mathrm{H}}_{2}{\mathrm{O}}_{2}\to \mathrm{ Polymer }+ {\mathrm{H}}_{2}\mathrm{O}$$

When H_2_O_2_ is added to the enzyme's native form, the enzyme produces an active intermediate compound, called compound 1 (Ci). One peroxidase substrate is oxidized by compound 1 to form compound II (Cii). After oxidizing a second peroxidase substrate, compound II produces another free radical product and returns to its native form as peroxidase. As a result of the polymerization of the free radicals, insoluble compounds have formed that precipitate from the solution [20]. These insoluble compounds are formed through a complex reaction involving the oxidation of two peroxidase substrates, leading to a unique enzymatic polymerization reaction.

Due to peroxidase's potential applications in various industries, these characteristics have attracted attention [16, 21]. Also, peroxidases that are already on the market, like horseradish peroxidase (HRP), *Bjerkandera adusta* peroxidase, and streptavidin peroxidase, are not likely to meet the growing need for peroxidases in industry. As a result, new sources of peroxidase are required to meet rising market demands [22].

Therefore, the present study was designed to isolate bacteria that degrade phenol in various wastewater and effluent sources. The isolate was screened for potential phenol degradation by this bacterial strain. Meanwhile, the optimization, purification, application of peroxidase production and evaluation of its properties were investigated. Further, the kinetic properties of the enzyme involved in phenol degradation were also assessed.

## Materials and methods

### Water samples collection

In this study, seven water samples were collected from different sites in El-Gharbya Governorate, Tanta, Egypt: car repair shop effluent, water treatment plant influent, wastewater treatment plant influent, petrochemical company cooling effluent, restaurant effluent, and car washing effluent. As soon as the water samples were collected, they were stored at 4 °C in 500-mL screw-cap glass bottles and immediately transported to the laboratory for further analysis [23].

### Strain enrichment and Isolation of peroxidase-producing strain

During enrichment culture, all samples of water were inoculated into MSM medium [MgSO_4_·7H_2_O, 0.1; NaCl, 0.2; NH_4_Cl, 0.5; Na_2_HPO_4_·12H_2_O, 0.5; KH_2_PO_4_, 0.5; FeCl_3_·6H_2_O, 0.1; and CaSO_4_·H_2_O, 0.1] [24], containing solely phenol as a carbon source. Phenol concentrations were increased from 770 to 1700 mg/L. Final enriched media were serially diluted and spread over LB agar plates [25]. Single colonies with morphological differences were selected from the incubated plates and streaked onto a new plate for further study. The morphological and Gram staining were evaluated for the phenol-degrading bacterial strains via Bergey's handbook of determinative bacteriology [26].

### Identification of the most promising peroxidase producer

There were 25 different isolates (encoded 1–25) that showed activity among 59 bacteria from different water samples, and 25 of them were chosen for further investigation. During the growth of the bacteria, periodic subcultures were performed every 2–3 weeks followed by 24 h of growth at 37 °C in a static incubator. Since isolates were primarily extracellular in nature, crude peroxidase was obtained from cell-free supernatant.

Out of those with peroxidase-positive reactions, the isolate that produced the most extracellular peroxidase was directed for further investigation. 16S rDNA sequencing was performed on bacterial strains using genomic DNA isolation, and PCR amplification. 16S rDNA sequences for the selected isolate were aligned with GenBank (http://blast.ncbi.nlm.nih.gov/Blast.cgi) using multiple-sequence alignment software, CLUSTAL W. Based on the homology of 16S rDNA sequences, a phylogenetic tree was constructed using MEGA 5.1 software to determine the taxonomy of the isolate using a neighbour-joining algorithm. Bootstrap analyses were also conducted with the Jukes-Cantor method to estimate distances [27]. An open-access database entry with accession number OP458197 contains the sequences of 16S rRNA genes amplified from the strain described in this study.

#### A qualitative analysis of peroxidase activity

In 100 ml of MSM medium, 0.02% of Poly-R478 and 0.01% w/v Azure B then 1.6% of agar are added along with 1 ml of 20% (w/v) glucose. Aseptically transferred medium is then seeded with bacterial strains and incubated at 27 °C in the dark. Observing a clear halo zone around the violet-colored test colony around screening plates for 10 days indicates the presence of phenol-degrading peroxidases as described previously [28, 29].

### Phenol degradation

We inoculated 2% of the final bacterial inoculum into MSM media containing phenol as the only carbon source. As a next step, the bacterial strain culture was prepared, and the optical density was determined at 600 nm. Phenol concentrations were raised from 770 mg/L to 1700 mg/L. Spectrophotometers at 600 nm were used to measure phenol concentrations following standard methods described by the American Public Health Association [30].

### Optimization of culture condition for production of extracellular peroxidase

Different physicochemical factors as well as nutritional requirements were examined systematically to determine how they may affect the production of extracellular peroxidase by the isolate. At predetermined time intervals, several factors affecting peroxidase production were manipulated one at a time, such as the incubation period (12–96 h), pH (5.0–8.0), and temperature (25–50 °C) [31]. Further, a variety of carbon and nitrogen sources were used for the optimization of peroxidase production. Each experiment was done three times, and the results were given as the average and the standard deviation (SD). In practice, 50 ml of nutrient broth (containing 0.2% yeast extract, 0.1% beef extract, 0.2% peptone, 0.5% sodium chloride, 1.0% glucose, and 0.05% hydrogen peroxide) was inoculated in 250 ml Erlenmeyer flasks with 8% (v/v) of 24-h-old seed culture. Using cell-free broth obtained after centrifuging at 10,000 × g for 10 min at 4 °C, peroxidase activity was determined 48 h after incubation at 37 °C under vigorous shaking (100 rpm) to determine the optimal conditions for the production of extracellular peroxidase by selected bacterial species. An assay method for measuring peroxidase activity was selected, and optimum conditions were established for further investigation.

### Protein determination

A stock solution of standard protein, bovine serum albumin at 1000 mg/mL, was prepared using the [32] method to determine enzyme concentration. Folin-Ciocalteu reagent was mixed with each sample for 30 min, and the absorbance was measured at 660 nm.

### Peroxidase purification

The crude enzyme was added to 2 g of ammonium sulphate until saturation was reached; it was left overnight at 4 °C and then centrifuged at 4000 g for 20 min. A minimum volume of 50 mM Tris–HCl buffer (pH 7.4) was dissolved in the precipitate, and the salts were dialyzed overnight at 4 °C. 0.05 M Tris–HCl buffer (pH 8.6) was pre-equilibrated with 0.05 M Tris–HCl buffer (pH 7.4) containing 0.1 M KCl and used for eluting the dialyzed fraction onto a Sephadex G-100 column (45 × 1.5 cm). The fractions that exhibited the highest levels of enzyme activity were lyophilized, and the resulting powder was stored at 4 °C [33].

### Polyacrylamide gel electrophoresis (PAGE)

Whole-cell lysates were generated by resuspending cells in 75 mL of lysis buffer (1 M Tris/HCl, pH 6.4), 10% sodium dodecyl sulfate (SDS), 5% glycerol, and 1% bromophenol blue (tracking dye). Then, SDS-PAGE was carried out by mixing the protein sample and 5 × loading buffer in a 4:1 (v/v) ratio and heating to 100 °C for 2–5 min. Wells were filled with samples and placed on gels. At a temperature of 8 °C and 100 V, electrophoresis was performed. After carefully removing the gel from the glass plates, it was stained with Coomassie Brilliant Blue R-250 and extensive de-staining was performed to visualize the bands of proteins [34]. De-stained gel images were captured and analyzed with software using a gel documentation system (Alpha InfoTech Corporation, USA).

### Kinetic properties of peroxidase

In a pH range of 5.0 to 10.0, the optimal pH for peroxidase activity was established. To determine pH stability, enzyme preparations at different pH values were incubated for 1 h, and then their relative activities were determined by standard assay methods. Using a peroxidase activity assay at different temperatures ranging from 30 to 100 °C, we determined the optimum temperature for enzyme activity. Moreover, peroxidase activity was measured after 1 h of incubation at the same temperature as the lyophilized enzyme. A substrate concentration was used to estimate peroxidase kinetic parameters (*Km* and *Vmax*). Based on the Linearweaver-Burk plots and an equation derived from the linear regression of the curve, the Michaelis–Menten parameters were calculated.

### Statistical analysis

one-way ANOVA was used to evaluate the significant differences. The significance level was set at *p* < 0.05. All data are presented as mean ± SD [35].

## Results

### Estimation of phenol levels in the samples

**Fig. 1:**
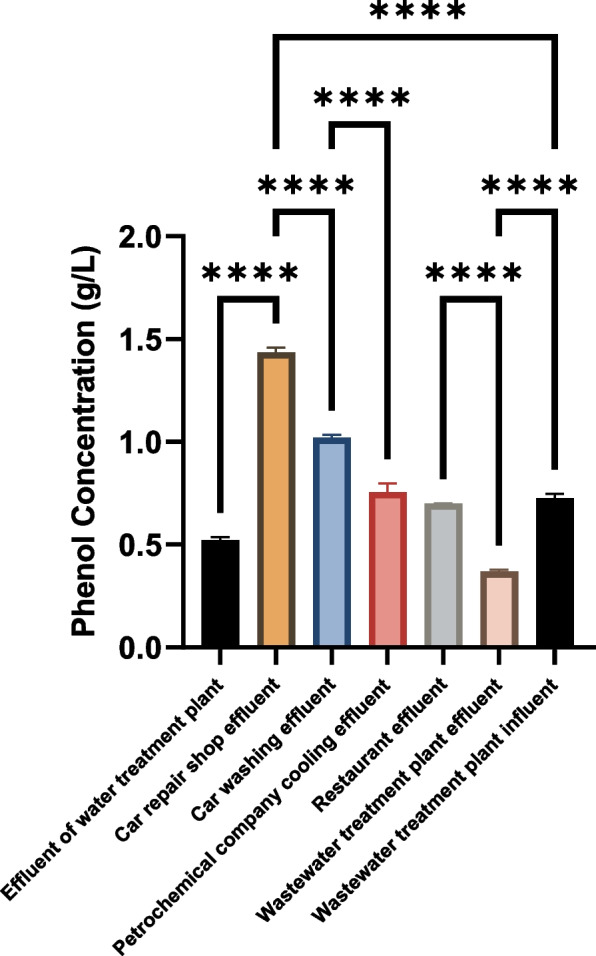
Phenol concentration (g/L) in different wastewater samples. The significance of the results was determined using a one-way ANOVA followed by a Tukey post-hoc test. Data are presented as mean ± SD ( **** (*P* ≤ 0.0001), *** (*P* ≤ 0.0002), and ** (*P* ≤ 0.0021), * (*P* ≤ 0.0332), (ns = 0.1234)

A total of seven different water samples were collected from different sites in El-Gharbya Governorate, Tanta, Egypt. As revealed in (Fig. 1), there were significant differences in the amount of phenol between the car repair shop effluent (1.42 ± 0.060 g/L) and the other samples. This suggests that the car repair shop effluent was the main contributor of phenol to the local environment, as it had the highest concentration of phenol compared to the other samples.

### Isolation and description of phenol-degrading strains

**Fig. 2:**
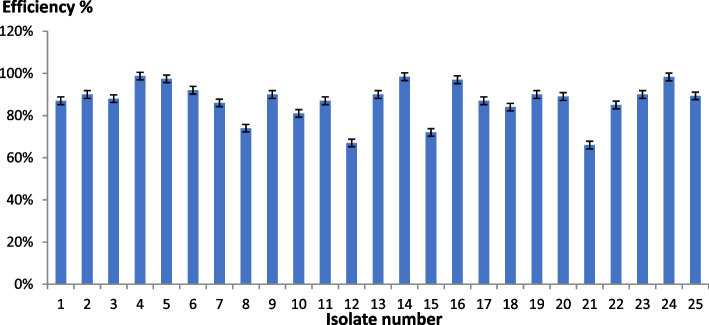
Efficiency of phenol-degrading strains on wastewater samples. Isolates No 1, 4,5,12,14,16,24 of the 25 isolates grew more than the others on LB agar plates with enrichment culture after 24 h of growth

The wastewater samples taken from the effluents were inoculated in a medium containing phenol for the enrichment and isolation of phenol-degrading bacteria. Three weeks of enrichment and one week of strain isolation yielded 25 isolates on LB agar plates with 100 mL of diluted enrichment culture after 24 h of growth. Six (Isolates no 4,5,12,14,16,24) of the 25 isolates grew more than the others on a medium containing phenol in all of these strains, all of which used phenol as their only carbon source (Fig. 2). The colony characteristics of the isolates were studied and recorded (Table S1). The exceptional isolate was designated as the phenol-degrading strain *Bacillus aryabhattai* B8W22 and used in subsequent studies.

### Identification of bacterial isolate by 16S rRNA gene

**Fig. 3:**
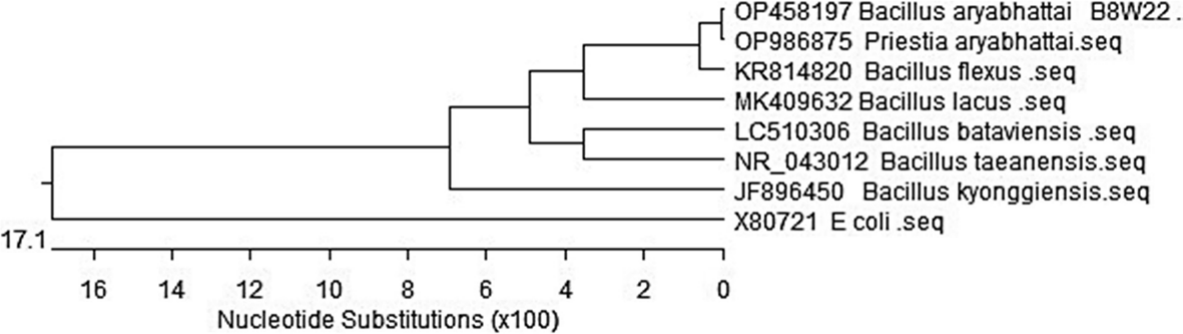
*Bacillus aryabhattai* B8W22 phylogenetic relationship with other published strains of the 16S rRNA gene

The sequences of the 16S rRNA gene were compared to the sequences of 16S rRNA regions in GenBank using a BLAST search of the National Center for Biotechnology Information (NCBI) databases for the selected isolate. The sequence homology retrieved from BLAST analysis with the NCBI data bank revealed that *Bacillus aryabhattai* B8W22 (16S ribosomal RNA gene, partial sequence) had a maximum identity of 99% with this bacterial isolate. The bootstrap consensus tree for the peroxidase-producing *Bacillus aryabhattai* B8W22 was constructed by aligning multiple sequences with the neighbour-joining method (Fig. 3). The accession number for the sequence information of the bacterial strain is OP458197.

### A qualitative analysis, phenol biodegradation and peroxidase activity

**Fig. 4:**
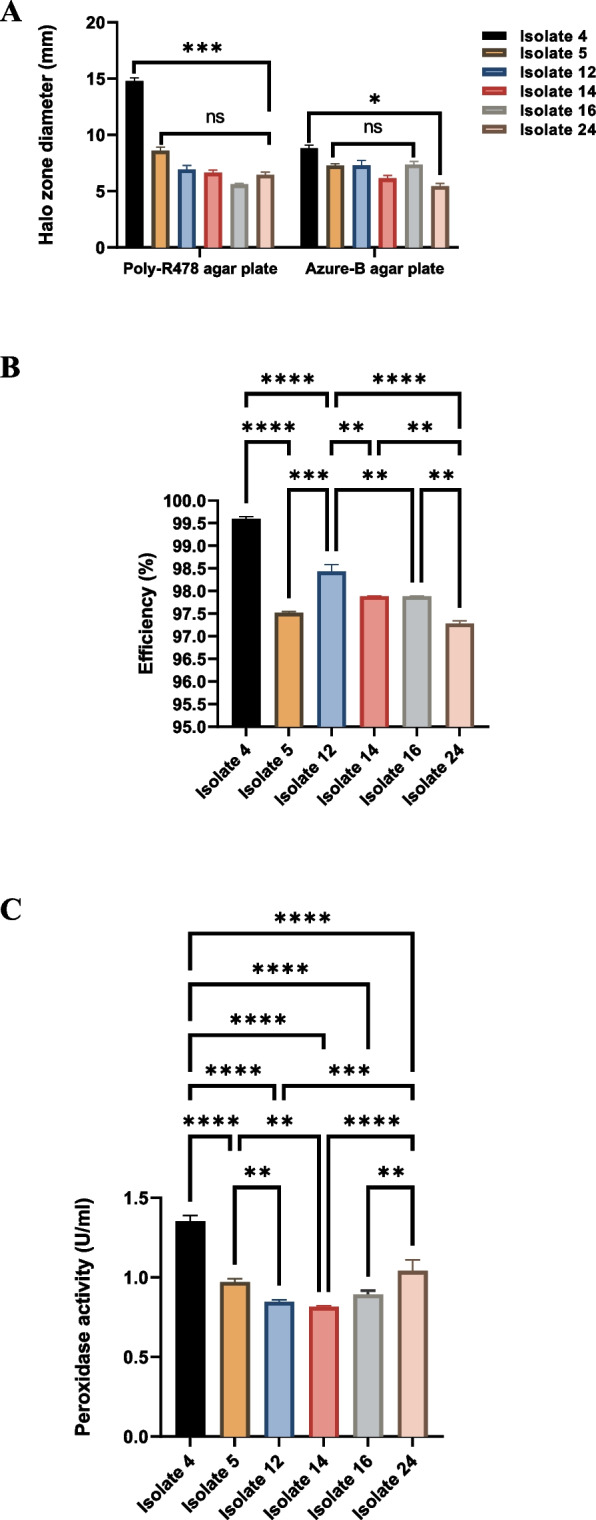
**A** Qualitative analysis of peroxidase by Poly-R478 and Azure-B plate assays. **B** *Bacillus aryabhattai* B8W22's phenol-degradation efficiency. Isolate No.4 showed a maximum biomass and phenol degradation efficiency of 99.6%. **C** Peroxidase activity among the selected isolates. Six different bacterial isolates were tested quantitatively for their ability to produce extracellular peroxidase enzymes. The data showed that each of the six isolates secreted peroxidase at a different level. However, isolate No. 4 had the highest peroxidase activity (U/ml). The significance of the results was determined using a one-way ANOVA followed by a Tukey post-hoc test. Data are presented as mean ± SD ( **** (*P* ≤ 0.0001), *** (*P* ≤ 0.0002), and ** (*P* ≤ 0.0021), * (*P* ≤ 0.0332), (ns = 0.1234)

Peroxidase activity was also assessed by evaluating how well the isolates utilized and degraded poly-R478 and Azure B. As seen in (Fig. 4A), each of the previous six isolates was able to utilize and degrade Poly-R478 as well as Azure B. The diameter of the halo zones indicated the degree of degradation by the isolates. Isolate No. 4 had the highest halo zones (Poly-R478: 14.79 ± 0.78 mm, Azure B: 8.81 ± 0.61 mm), whereas isolate No. 16 had the lowest poly-R478 degradation zone and isolate No. 24 had the lowest Azure B degradation zone. This indicates that there is a wide range of abilities among the isolates when it comes to degrading the two compounds. Isolate No. 4 was the most efficient at degrading both compounds, while isolate No. 16 was the least effective at degrading Poly-R478 and isolate No. 24 was the least effective at degrading Azure B. By monitoring phenol concentrations and cell growth at OD600 periodically, *Bacillus aryabhattai* B8W22's phenol-degradation efficiency and biomass were determined at various initial concentrations of phenol (200–2500 mg/L). Isolate No.4 showed a maximum biomass and phenol degradation efficiency of 99.6% (Fig. 4B). Concerning the enzyme activity, there were significant differences between all isolates with the highest peroxidase activity detected in isolate No.4 (1.34 ± 0.02 U/ml). The other bacterial isolates showed an extracellular peroxidase activity ranging from (0.82 to 1.05 U/ml.) (Fig. 4C).

### Effect of incubation time, temperature, and pH on peroxidase enzyme production

**Fig. 5:**
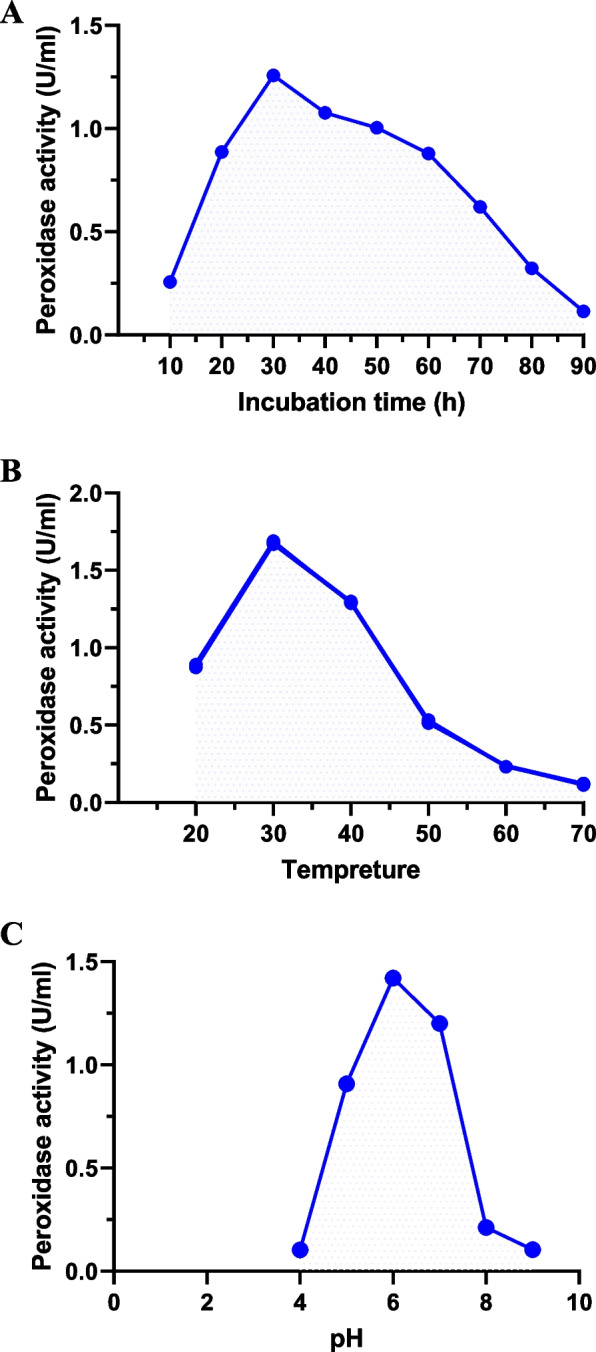
**A** Effect of incubation time on peroxidase enzyme production. **B** Effect of temperature on peroxidase enzyme production. **C** Effect of pH on peroxidase enzyme production

The peroxidase production by *Bacillus aryabhattai* B8W22 was determined at different incubation times. It was found that the activity was highly significant and increased until it reached its maximum at 30 h of incubation, then decreased to reach its minimum after 90 h (Fig. 5A). Furthermore, the effect of different temperatures (20–70 °C) on peroxidase production by *Bacillus aryabhattai* indicated that the highly significant maximum peroxidase activity was reached at 30 °C and was considerably reduced at 50 °C (Fig. 5B). Additionally, it was found that *Bacillus aryabhattai* B8W22 produced high amounts of peroxidase with an increasing pH of the production broth up to 6. However, a decrease in production was observed at a higher pH. The pH of 6 was therefore considered optimal for *Bacillus aryabhattai* B8W22 to produce extracellular peroxidase (1.43 U/ml) after incubation at 37 °C (Fig. 5C).

### Effect of carbon and nitrogen sources on peroxidase enzyme production

**Fig. 6:**
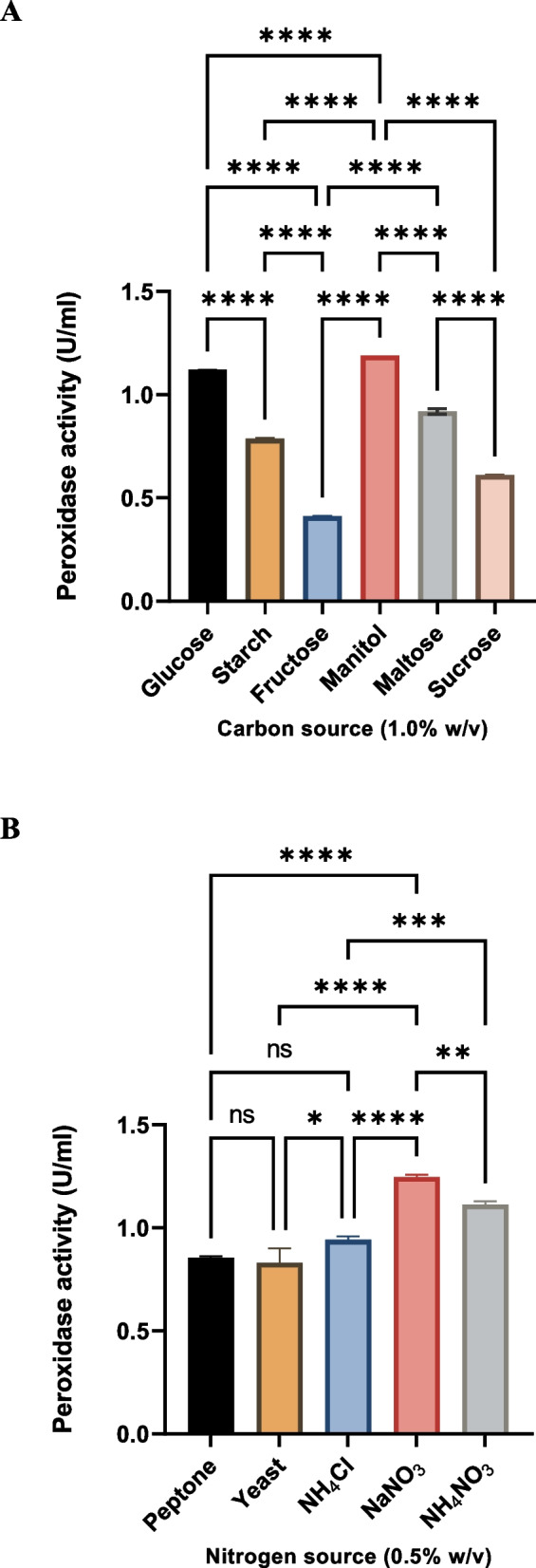
**A** Effect of carbon source on peroxidase production by *Bacillus aryabhattai* B8W22. **B** Effect of nitrogen source on peroxidase production by *Bacillus aryabhattai* B8W22. The significance of the results was determined using a one-way ANOVA followed by a Tukey post-hoc test. Data are presented as mean ± SD ( **** (*P* ≤ 0.0001), *** (*P* ≤ 0.0002), and ** (*P* ≤ 0.0021), * (*P* ≤ 0.0332), (ns = 0.1234)

Peroxidase production was assessed by investigating how the *Bacillus aryabhattai* B8W22 isolate utilized glucose, starch, fructose, manitol, maltose, and sucrose as carbon sources. It was evident that the carbon source that was used affected peroxidase production, with mannitol being the most preferred carbon source (1.24 ± 0.02 U/ml). Fructose and sucrose were observed to produce the least peroxidase when used as carbon sources, reaching 0.4 U/ml and 0.6 less than optimal values, respectively (Fig. 6A). Different nitrogen sources were examined for their effect on peroxidase production, including peptides, yeast extract, ammonium chloride, sodium nitrate, and ammonium nitrate. As a nitrogen source, *Bacillus aryabhattai* B8W22 can utilize sodium nitrate as a source of nitrogen, and there is a favourable response to the nitrate concentration, which leads to higher enzyme production at 1.31 U/mL. No significant change in enzyme production was observed with yeast extract or peptone, but sodium nitrate and ammonium nitrate significantly increased enzyme production between 1.2 and 1.31 U/mL (Fig. 6B).

### Purification of peroxidase from *Bacillus aryabhattai* B8W22

Ammonium sulphate precipitation, dialysis, and filtration chromatography that were used to purify peroxidase from *Bacillus aryabhattai* B8W22 were depicted in (Table 1). Illustratively, purification boosts enzyme-specific activity and purity while lowering total protein, total activity, and yield. In the crude enzyme, there were 182 mg of protein and 2.2 U of peroxidase activity, and the specific enzyme activity was 0.012 U/mg. In addition, the enzyme was purified 2.33-fold to yield 53% of the enzyme after it was purified from 42 mg of protein with 1.2 U of enzyme activity.

**Table 1:** Purification procedure of peroxidase from *Bacillus aryabhattai* B8W22

	**Total activity** **U**	**Total protein** **mg**	**Specific activity** **U/mg protein**	**Purification fold**	**Recovery** **%**
**Crude**	2.2	182	0.012	1	**100**
**Ammonium sulfate precipitation**	1.87	99	0.018	1.5	**84**
**Dialysis**	1.80	95	0.0189	1.57	**83.4**
**Sephadex G-25**	1.2	42	0.028	2.33	**53**

### SDS-PAGE and molecular weight estimation

**Fig. 7:**
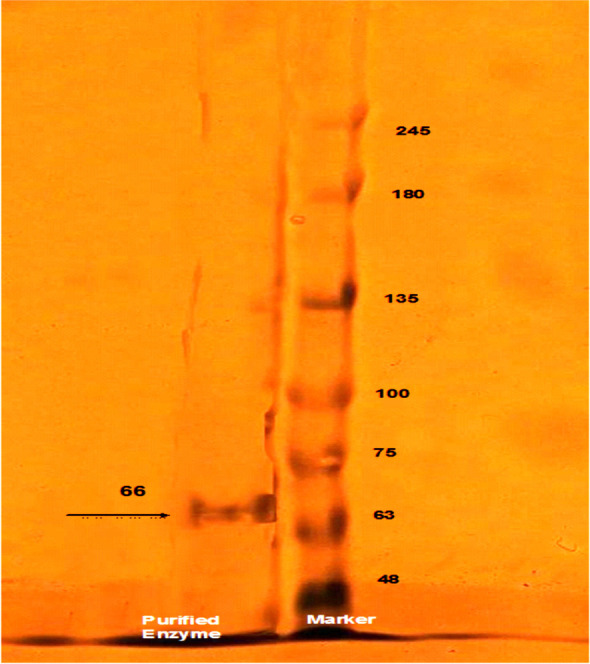
SDS–polyacrylamide gel electrophoresis of the purified peroxidase enzyme from *Bacillus aryabhattai* B8W22

Purified peroxidase was assessed for homogeneity and molecular weight by sodium dodecyl sulfate–polyacrylamide gel electrophoresis (SDS-PAGE). Peroxidase preparations were found to be generally pure, as only one distinct protein band with a 66 kDa apparent molecular weight was observed compared to standard molecular weight markers (Fig. 7).

### Kinetic properties of the purified peroxidase

**Fig. 8:**
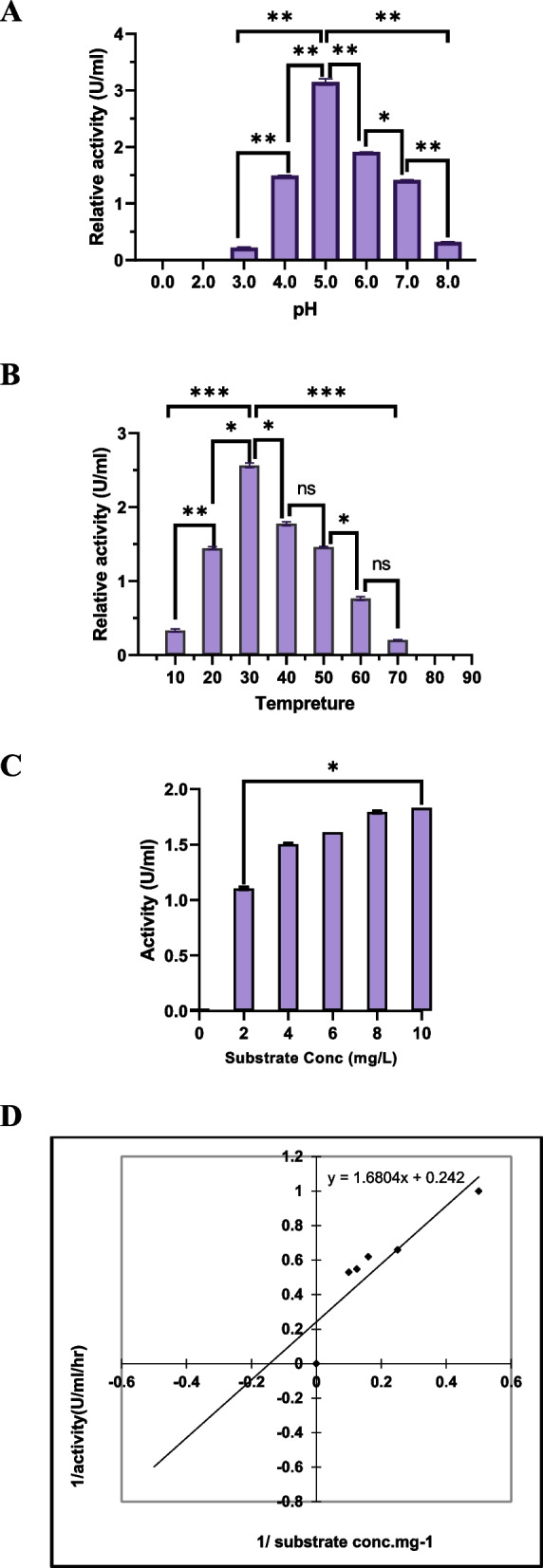
**A** Effect of pH on the activity and stability of peroxidase. **B** Effect of temperature on the activity and stability of peroxidase. **C** Effect of substrate concentration on the activity of peroxidase from *Bacillus aryabhattai B8W22*. **D** Lineweaver–Burk plot for *Bacillus aryabhattai* B8W22 peroxidase under varying guaiacol concentrations indicating the *Vmax* and *Km* values. Data are presented as mean ± SD ( **** (*P* ≤ 0.0001), *** (*P* ≤ 0.0002), and ** (*P* ≤ 0.0021), * (*P* ≤ 0.0332), (ns = 0.1234)

Over a broad pH range, the enzyme was active, with its activity gradually rising to pH 5 (Fig. 8A). At pH values below optimal, enzymatic activity could be retained up to 0.3 U/mL and 1.5 U/mL at pH values of 8.0 and 4.0, respectively. Purified peroxidase exhibits its maximum activity at 30 °C (2.6 U/mL), with its activity gradually decreasing above this temperature (Fig. 8B). Over a temperature range of 30–40 °C, the peroxidase displayed thermostability. The lowest activity was observed at 70 °C. A *Km* value for an enzyme is dependent both on the substrate and on the environmental conditions at the time of kinetic measurement. These conditions include temperature, pH, and ionic strength. According to Michaelis–Menten behaviour, the kinetic constants of purified peroxidase of *Bacillus aryabhattai* B8W22 have been determined at a concentration of substrate guaiacol (Fig. 8C). *Km* and* Vmax* values were calculated using the Lineweaver–Burk plot between 1/V and 1/[S]. A *Bacillus aryabhattai* B8W22 peroxidase was found to have a *Km* and a *Vmax* of 6.942 mg/ml and 4.132 µmole/ml/hr, respectively using guaiacol as a substrate in (Fig. 8D).

**Fig. 9:**
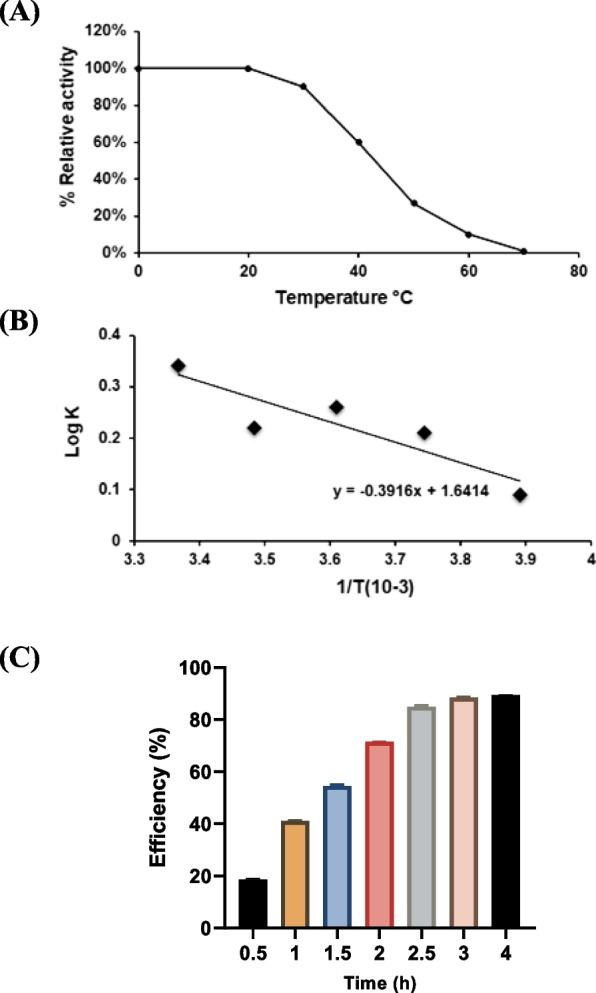
**A** Thermal stability of peroxidase from* Bacillus aryabhattai* B8W22. **B** Arrhenius plot to calculate activation energy (*Ea*) of peroxidase from* Bacillus aryabhattai* B8W22*.* **C** The efficiency of purified peroxidase of *Bacillus aryabhattai* B8W22 on phenol removal from synthetic water

In (Fig. 9A), the peroxidase enzyme exhibited 100% relative activity over a temperature range of 10–20 °C. The results of this study indicate that peroxidase has high thermal stability, with about 10% of its relative activity remaining at 60 °C and gradually decreasing to 1% at 70 °C. Peroxidase has been calculated to have activation energy between 20 °C and 70 °C. In the temperature range mentioned previously, the Arrhenius plots indicated a linear relationship between Ea and temperature. For guaiacol, the activation energy (Ea) was calculated as 2.27 kcal/mol (Fig. 9B). In terms of phenol removal from synthetic wastewater, (Fig. 9C) showed that the phenol concentration was highly reduced after synthetic wastewater treatment with purified peroxidase enzyme. After 4 h of incubation, the purified enzyme was able to remove 95% of the phenol.

## Discussion

Various microbial species are more adaptable and capable of rehabilitation in contaminated environments when exposed to phenol [36, 37]. Bacteria have gained popularity as an eco-friendly and low-cost method of dealing with environmental pollutants [38]. Thus, finding new bacteria that break down phenol is crucial for the bioremediation of ecosystems that have been impacted by phenol. Therefore, we aimed to exploit the enzymatic potential of *Bacillus aryabhattai* B8W22, which was originally isolated from water samples collected from different locations in Egypt.

Initial testing revealed extracellular peroxidase activity in 25 of 59 bacterial isolates obtained from different wastewater samples. Among the isolates obtained from effluent-polluted water samples, isolate No.4 displayed maximum extracellular peroxidase activity (0.33 ± 0.01 U/ml) at 37 °C, hence it was selected for further study. As part of the oxidative stress defence system, peroxidase breaks down substances that contain the peroxidic bond, preferentially H_2_O_2_ [39]. Nyanhongo et al. [40] reported that peroxidases can use H_2_O_2_, methyl hydrogen peroxide, and ethyl hydrogen peroxide as oxidizing agents. Furthermore, microorganisms survive oxidative stress by regulating the activity of peroxidase [41] and catalase [42]. This may explain *Bacillus aryabhattai* B8W22's production of extracellular peroxidase from wastewater samples containing high levels of oxidative stress.

Among the isolates included in our study, (isolate No.4) showed the highest phenol degradation efficiency of 98.7%. By increasing the phenol concentration to 97.2%, such high levels of degradation efficiency were achieved. These results indicate that this particular isolate is well suited to handling high levels of phenol in aqueous environments. Furthermore, the increase in phenol concentration did not have a negative effect on the isolate's degradation efficiency, suggesting that it is capable of handling even greater concentrations of phenol. Therefore, the findings of this study indicate that the isolated strain of bacteria can tolerate and break down significant concentrations of phenol. As a result, it can be used as a reliable method of wastewater treatment, particularly when the phenol concentration is high. These findings agreed with a previous study that reported that few strains have been found to be capable of decomposing phenol at a concentration of 1000 mg/L [43]. Also, in the study by Bevilaqua et al., phenol was removed by biological and enzymatic methods [44]. A previous study reported that *Pseudomonas putida* strains were the best phenol-degrading bacteria capable of consuming 500–600 mg/L phenol after 48 h of incubation. Therefore using bacterial isolate can reduce treatment time while also increasing the rate of phenol bioremediation up to 700 mg/l [45].

Based on 16S rRNA gene sequencing, isolate No.4 was identified as *Bacillus aryabhattai* B8W22. Among the most important industrial microbes, *Bacillus* species are known for their ability to produce huge quantities of extracellular proteins, rapid growth rate, and overall safety [46].

Our results exhibited that *Bacillus aryabhattai* B8W22 peroxidase activity was increased to its maximum level after 24 h of incubation, then decreased to the minimum level after 48 h, which could be attributed to the presence of byproducts that inhibited enzyme production. According to Falade et al. [22], the ideal assembly of exoperoxidase by *E. adhaerens* NWODO-2 occurred after 48 h of incubation. In contrast, this result contradicts similarly conducted studies [47] indicating that the maximum peroxidase was synthesised at 72 h.

In this study, *Bacillus aryabhattai* B8W22 was tested for the optimal temperature for producing peroxidase. At 30 °C, peroxidase production increased and reached its peak activity, whereas it diminished after being incubated at 50 °C. A similar finding was reported by Rajkumar et al. [48], who discovered that *Bacillus* sp. produces its finest peroxidase at 30 °C. In contrast, Rao et al. [49] suggested that *Bacillus subtilis* produces the most peroxidase at 37 °C.

The cultivation pH has a considerable impact on microbial metabolism and growth because the charge on bacterial cells determines their ability to absorb nutrients [50]. So, it is crucial to determine the pH of the medium that is most conducive to bacterial metabolic processes. This result aligns with the results of an earlier study by Falade et al. [51], which reported that *Bacillus* sp. produces peroxidase best at pH 8. Nevertheless, Rao et al. [49] confirmed that pH 6 promoted extreme peroxidase production by *Bacillus subtilis*.

Further, our results showed that NaNO_3_ was a significant enzyme synthesis enhancer, but peptone was an inhibitor. Several carbon sources have been found to affect bacterial enzyme synthesis [52]. In the present study, glucose and mannitol were the only carbon sources that increased enzyme production, whereas all other carbon sources inhibited it. Similar findings were reported by Sadhu et al. [53], who investigated the production of peroxidase by bacteria using eight different sources of carbon.

Purification and characterization of peroxidase(s) from *Bacillus aryabhattai* B8W22 were attempted. The elution profile showed a single peak indicative of peroxidase activity. According to the results, peroxidase was effectively purified by 2.33 folds. As a result of fractional purification, contaminating enzymes are removed, and specific activity is increased from 0.012 to 0.028 U/mg (Sephadex G-25). In addition, Kalyani et al. [54] also used gel filtration chromatography to purify fractions with high peroxidase activity from *Pseudomonas* sp. Elsayed et al. [55] demonstrated the effectiveness of these techniques using *Pseudomonas* sp.

SDS-PAGE analysis was performed on the pooled active fractions following gel filtration chromatography. One pure protein band corresponds to purified peroxidase, which has a molecular weight (MW) between 63 and 75 kDa. The precise MW of purified peroxidase was determined at 66 kDa by the SDS-PAGE method. Changes in amino acid sequences or glycosylation levels could explain the changes in peroxidase molecular weight. Peroxidase is generally believed to have a molecular mass of 15–40 kDa in microorganisms [56]. Similarly, in SDS-PAGE, Roa et al. [49] found a single band with a molecular mass of 44 kDa in the isolated enzyme from *Bacillus subtilis*. It has been reported in previous studies that peroxidases from bacterial sources purified from *Acinetobacter calcoaceticus* NCIM 2890, *Bacillus* sp. VUS, *Pseudomonas* sp. SUK1 and *Klebsiella pneumoniae* contains molecular weights of 110 kDa, 43 kDa, 86 kDa, and 48 kDa, respectively [57, 58].

Further characterization of peroxidase purified from *Bacillus aryabhattai* B8W22 was carried out. Based on the results obtained, the peroxidase activity increased with increasing substrate concentration in the presence of a given amount of enzyme until a certain concentration. Further increases in substrate concentration didn't have a significant effect on enzyme activity. A possible explanation is that all enzyme molecules are saturated with the substrate at the same time [59]. A Lineweaver–Burk plot was used to calculate the kinetic parameters *Km* and *Vmax* of the peroxidase produced from *Bacillus aryabhattai* B8W22. These values are 6.942 mg/ml and 4.132 mol/ml/hr, respectively. *Km* represents the degree to which an enzyme interacts with its substrate; a lower *Km* value indicates the enzyme has a high affinity for its substrate, while a higher *Vmax* value indicates that only a minimal amount of enzyme is required to convert the substrate. A study by Asgher et al. [60] reported a *Km* value of 1.27 mM for hydrogen peroxide and a *Vmax* value of 0.138 u/ml/min for hydrogen peroxide and peroxidase from *Raphanus sativus*. According to Rayan et al. [61], the calculated Km values for peroxidase from *Granada Clingstone* peaches were 5.15 mM guaiacol; and 2.47 mM guaiacol when used as substrates for wheat peroxidase [62].

It has been reported by Pinto et al. [63] that peroxidases can oxidize several substrates in the presence of hydrogen peroxide. Peroxidase had a high affinity and catalytic efficiency for O-dianisidine. Differences in substrate affinity could be explained by the redox potential of this enzyme and its contribution to the breakdown of refractory chemicals found in contaminated soils or residual wastewater [64]. As a result of the existence of isoenzymes with dissimilar levels of temperature confrontation [65], thermostability cannot be applied to peroxidases.

## Conclusion

To conclude, the bacterium isolated from wastewater effluents in Egypt was capable of degrading phenol. By analysing the 16S rDNA sequence and phylogenetic tree, *Bacillus aryabhattai* B8W22 was identified. *Bacillus aryabhattai* B8W22 is capable of growing in liquid media containing phenol as its only carbon and energy source. The bacterial peroxidase was purified to homogeneity using column chromatography. The purified peroxidase possessed a molecular weight of 66 kDa in its native form. The strain thrived best at 30 °C and pH 5.8 for phenol degradation. Considering native microorganisms were more adaptable to polluted environments than non-indigenous microbes, their predominance allowed phenol contamination to be bioremediated. A strain of *B*. *aryabhattai* B8W22 isolated from El-Gharbia, Egypt, may be useful for the bioremediation of phenol contamination.

## Supplementary Information

**Additional file 1: Table S1. **Morphological characterizations and Gram stain of the twenty-five bacterial isolates.

## Data Availability

The datasets generated and/or analysed during the current study are available in the GeneBank repository with accession number OP458197.
