# Supplementary material for: Evaluation of Bacillus aryabhattai B8W22 peroxidase for phenol removal in waste water effluents
Source: BMC Microbiol. 2023 Apr 29;23:119. doi: 10.1186/s12866-023-02850-9 (PMC10148497; doi:10.1186/s12866-023-02850-9)
Supplement: Supplementary file 1 — Additional file 1: Table S1. Morphological characterizations and Gram stain of the twenty-five bacterial isolates. [file 12866_2023_2850_MOESM1_ESM.docx]

**Table S1:** Morphological characterizations and Gram stain of the twenty-five bacterial isolates**.**

| Isolate  No | Shape of colonies | | | | | Shape of cells | Gram reaction |
| --- | --- | --- | --- | --- | --- | --- | --- |
|  | **Colour** | **Form** | **Elevation** | **Margin** | **Surface** |  |  |
| 1 | White | circular | Flat | Entire | Smooth | Rod shape | +ve |
| 2 | Yellow | circular | Convex | Entire | Smooth | Coccus | +ve |
| 3 | Buff | circular | Convex | Entire | Smooth | Coccus | -ve |
| 4 | Off white | circular | Flat | Entire | Smooth | Rod shape | +ve |
| 5 | Buff | irregular | Convex | Entire | Smooth | Rod shape | -ve |
| 6 | Off white | irregular | Raised | Erose | Smooth | Rod shape | +ve |
| 7 | Brown | irregular | Convex | Entire | Smooth | Rod shape | +ve |
| 8 | White | circular | Raised | Lobate | Smooth | Coccus | +ve |
| 9 | Orange | circular | Flat | Entire | Smooth | Rod shape | +ve |
| 10 | Off white | irregular | Flat | Lobate | Smooth | Coccus | +ve |
| 11 | Off white | circular | Flat | Entire | Smooth | Rod shape | -ve |
| 12 | Yellow | circular | Flat | Entire | Smooth | Coccus | +ve |
| 13 | Yellow | irregular | Flat | Undulate | Smooth | Rod shape | -ve |
| 14 | Off white | irregular | Umbonate | Lobate | Smooth | Rod shape | +ve |
| 15 | Off white | circular | Raised | Entire | Smooth | Rod shape | +ve |
| 16 | Off white | irregular | Flat | Entire | Smooth | Coccus | +ve |
| 17 | Off white | circular | Umbonate | Undulate | Smooth | Rod shape | -ve |
| 18 | Brown | circular | Flat | Entire | Smooth | Coccus | +ve |
| 19 | Pale brown | circular | Flat | Entire | Smooth | Rod shape | -ve |
| 20 | Pale yellow | irregular | Umbonate | Lobate | Smooth | Coccus | +ve |
| 21 | Yellow | irregular | Umbonate | Curled | Smooth | Smooth | -ve |
| 22 | Off white | circular | Convex | Entire | Smooth | Smooth | +ve |
| 23 | Off white | irregular | Flat | Curled | Smooth | Smooth | +ve |
| 24 | Off white | circular | Flat | Entire | Smooth | Smooth | +ve |
| 25 | Off white | irregular | Flat | Curled | Smooth | Smooth | +ve |
